# Identification and Validation of Glycosyltransferases Correlated with Cuproptosis as a Prognostic Model for Colon Adenocarcinoma

**DOI:** 10.3390/cells11233728

**Published:** 2022-11-22

**Authors:** Wei Ma, Lingyuan Zhu, Shushu Song, Bo Liu, Jianxin Gu

**Affiliations:** NHC Key Laboratory of Glycoconjugates Research, Department of Biochemistry and Molecular Biology, School of Basic Medical Sciences, Fudan University, Shanghai 200032, China

**Keywords:** cuproptosis, glycosyltransferase, colon adenocarcinoma, prognosis, prediction

## Abstract

Cuproptosis is a newly defined programmed cell death pattern and is believed to play an important role in tumorigenesis and progression. In addition, many studies have shown that glycosylation modification is of vital importance in tumor progression. However, it remains unclear whether glycosyltransferases, the most critical enzymes involved in glycosylation modification, are associated with cuproptosis. In this study, we used bioinformatic methods to construct a signature of cuproptosis-related glycosyltransferases to predict the prognosis of colon adenocarcinoma patients. We found that cuproptosis was highly correlated with four glycosyltransferases in COAD, and our model predicted the prognosis of COAD patients. Further analysis of related functions revealed the possibility that cuproptosis-related glycosyltransferase Exostosin-like 2 (*EXTL2*) participated in tumor immunity.

## 1. Introduction

The incidence of colon adenocarcinoma (COAD) is increasing year by year. Colon adenocarcinoma accounts for about 10% of all cancers and is the second most common cause of cancer death [1]. It is the third most common cancer in men and the second most common cancer in women [2]. The prognosis of colon adenocarcinoma is of great concern, but many factors can affect the patient’s prognosis, such as TNM stage, clinical factors, pathological factors, biological factors, and so on. Studies have shown that about 20% of colon adenocarcinoma patients have metastases at diagnosis, and up to half of the patients who initially have localized disease will have metastases [3]. The establishment of a prognosis model for colon adenocarcinoma is of great significance for the selection and optimization of treatment methods.

Cuproptosis is a novel type of cell death that differs from known cell death mechanisms such as apoptosis, autophagy, and ferroptosis [4]. It was first reported by Tsvetkov et al. that excessive accumulation of intracellular copper direct binds to lipoacylated components of the tricarboxylic acid cycle, which leads to aggregation of lipoacylated proteins and destabilizes Fe-S cluster proteins, eventually leading to proteotoxic stress and eventual cell death. Copper is essential for life processes such as energy metabolism, reactive oxygen species detoxification, iron uptake, and signaling in eukaryotes. Mitochondria collect copper for the assembly of copper enzymes [5]. A copper imbalance has been linked to some diseases, including anemia, neutropenia, thrombocytopenia, and tumor development [6,7,8]. Consumption of copper causes metabolic reprogramming from oxidative to glycolytic metabolism and lowers energy generation, which in turn increases tumor angiogenesis [9,10]. Abnormal copper accumulation in cancer cells can be a target for new chemotherapeutic agents, and drugs targeting copper have been utilized for clinical treatment in cancer [11,12]. Copper may play a role in the etiology and progression of cancer. Protein post-translational modifications (PTMs) can change the stability, activity, and cellular localization of proteins, which is an important way to regulate protein function.

The most common post-translational modification in the body is glycosylation, and it is estimated that more than half of all proteins are glycosylated [13]. Changes in the glycosylation pathway actively drive the malignant phenotype of cancer, and glycosylation changes can be detected in the tissues and biological fluids of cancer patients, where these changes have utility as disease biomarkers [14,15,16]. The glycosylation of proteins is inseparable from the action of various glycosyltransferases. Based on sequence similarity, glycosyltransferases have been classified into 78 families (GT family) [17]. Another important enzyme involved in the alteration of glycosylation is glycosidase. It has been shown that glycosidases are higher in tumor interstitial fluid and tumor-bearing animal and human serum. At present, the identification, evolution, and function of glycosyltransferases are still far from being understood and deserve further exploration [18].

However, cuproptosis-related glycosyltransferase in colorectal cancer patients has not been reported as a biomarker. In this study, based on the TCGA database, the prognostic model of COAD was constructed, and several related glycosyltransferases were identified as potential biomarkers. In addition, we conducted a comprehensive analysis of risk models for functional enrichment, drug resistance, immunotherapy, immune infiltration, and somatic mutations.

## 2. Materials and Methods

### 2.1. Data Source

In this study, we downloaded transcriptome data and clinical data from the TCGA database (https://portal.gdc.cancer.gov, accessed on 15 June 2022). The transcriptome data included 398 tumor tissues and 39 normal colon tissues. The clinical data contained information such as age, sex, survival time, survival status, pT stage, pN stage, and pM stage. We selected the clinical and sample information of 367 patients and randomly divided them into training and test groups using R software (version 4.1.3; creator Ross Ihaka and Robert Gentleman, Vienna, Austria).

### 2.2. Recognition of Cuproptosis-Related Glycosyltransferase

To seek cuprotosis-related glycosyltransferases, we retrieved 186 glycosyltransferases from a research article [19]. Nineteen cuprotosis-related genes were identified from a literature review [20]. To find a correlation between cuprotosis-related genes and glycosyltransferases, we used Pearson’s correlational analysis to investigate.

### 2.3. Biometric Analysis

We first used univariate COX regression analysis to screen for cuprotosis-related glycosyltransferases associated with prognosis; then, we used LASSO regression to build prognostic models and prevent overfitting. Next, we established cuprotosis-related glycosyltransferases for final inclusion in the model by multivariate COX regression analysis. Principal component analysis (PCA) uses dimensionality reduction to discern potential differences between high-risk and low-risk groups.

### 2.4. Cuproptosis-Related Glycosyltransferase Functional Analysis

Functional analysis of differential genes in high- and low-risk groups was performed by using GO and KEGG enrichment analysis, mainly including the analysis of biological processes (BP), cell components (CC), molecular functions (MF), and biological pathway information. Next, we analyzed the immune function of the differential genes. We analyzed their potential function by using STRING: functional protein association networks (https://string-db.org, accessed on 10 July 2022) to predict the interacting proteins of four glycosyltransferases.

### 2.5. Search for Potential Drugs for the Disease

The drug sensitivity of patients with different risk groups was assessed using the R “pRRophetic” package, which predicts the 50% inhibitory concentrations of chemotherapy drugs common to colon cancer (IC50). We used the Wilcoxon Signature Level Test to evaluate the differences between groups.

## 3. Results

### 3.1. Exploring Cuproptosis-Related Glycosyltransferases with Prognostic Value in Patients with COAD

**Figure 1:**
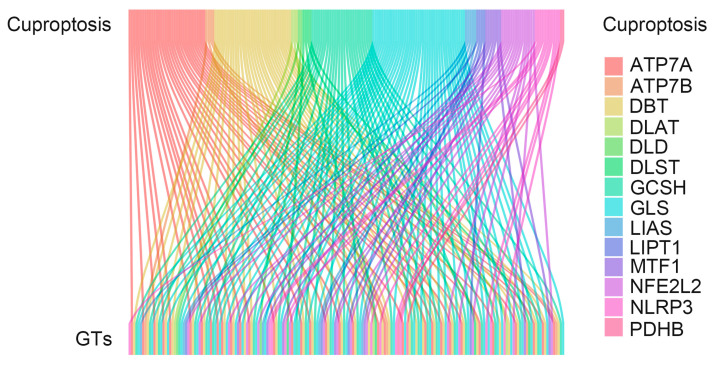
The correlation of cuproptosis-related genes with glycosyltransferases using the Sankey diagram.

**Figure 2:**
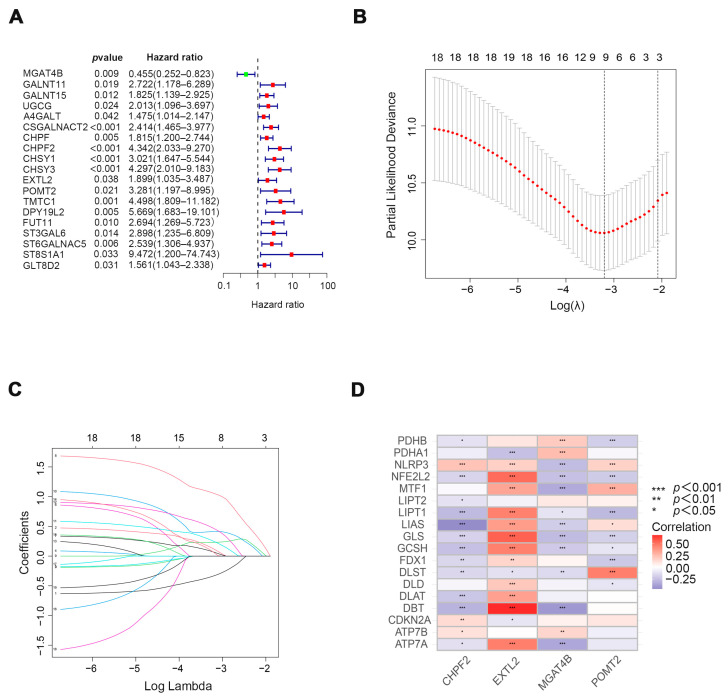
Four prognostically significant cuproptosis-related glycosyltransferases were obtained. (**A**) Nineteen glycosyltransferases with prognostic significance were identified by univariate COX analysis. (**B**) Using the minimum criterion method, the confidence intervals for each are shown and dotted with the best value source. (**C**) Partial likelihood deviance in the case of different numbers of variables. The horizontal axis represents the log value of the independent variable lambda, and the vertical axis represents the coefficient of the independent variable. (**D**) A heatmap depicts the relationship between four glycosyltransferases and the cuproptosis-related genes. * *p* < 0.05, ** *p* < 0.01, *** *p* < 0.001.

In the study, we extracted 19 cuproptosis-related genes and 186 glycosyltransferases from The Cancer Genome Atlas (TCGA) database COAD array. Co-expression analysis of glycosyltransferase and cuproptosis-related genes was performed to explore the correlation between them. Sankey’s diagram was used to demonstrate their correlation (Figure 1). To further explore the relationship between cuproptosis-related glycosyltransferases and the survival of COAD patients, we identified 19 prognostic glycosyltransferases (Figure 2A) using univariate COX regression analysis. Nine cuproptosis-related glycosyltransferase genes (*MGAT4B*, *GALNT11*, *CHPF*, *CHPF2*, *CHSY1*, *CHSY3*, *EXTL2*, *POMT2*, and *DPY19L2*) were selected using LASSO regression analysis (Figure 2B,C). Multivariate COX regression analysis identified four cuproptosis-related glycosyltransferase genes (*CHPF2*, *EXTL2*, *MGAT4B*, and *POMT2*) (Figure 2D). These results suggest that cuproptosis-related glycosyltransferases may be utilized to predict the prognosis of patients with COAD.

### 3.2. Evaluation and Validation of Prognostic Models

**Figure 3:**
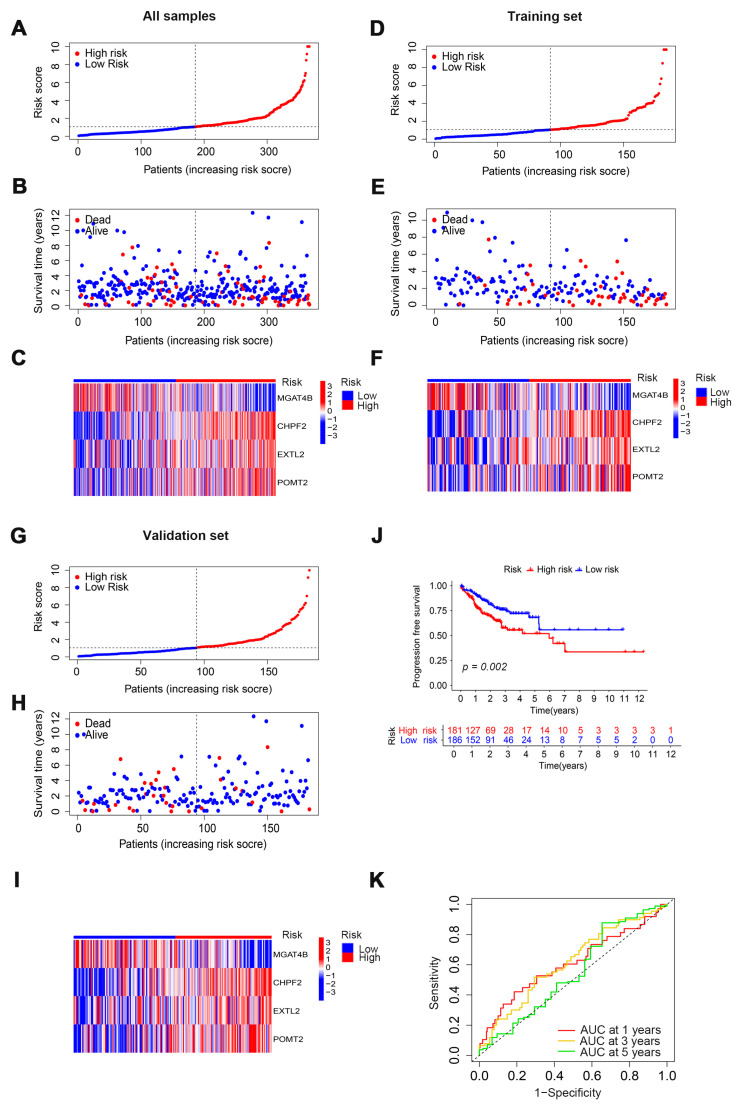
Validation of the prognostic predictive ability of cuproptosis-related glycosyltransferase. Prognostic characteristics in COAD for risk scores in the high-risk and low-risk groups for all samples (**A**), training set (**D**), and validation set (**G**). Survival status of the high and low-risk groups in all samples (**B**), training set (**E**), and validation set (**H**). Expression of four cuproptosis-related glycosyltransferases in all samples (**C**), training set (**F**), and validation set (**I**). (**J**) Analysis of progression-free survival in high-risk and low-risk groups for all samples. (**K**) ROC curves with prognostic characteristics in all samples. ROC, receiver operating characteristics; AUC, area under the curve.

We established a prognostic model by using the results of the multivariate COX regression analysis to verify the prognostic ability of four cuproptosis-related glycosyltransferase genes (*CHPF2*, *EXTL2*, *MGAT4B*, and *POMT2*). We first calculated the risk score for all patients and divided them into high and low-risk groups according to the median risk score; then, we randomly assigned all patients in the high and low-risk groups to the training group and the test group (Figure 3A,D,G). We found that the high-risk group had a higher death rate than the low-risk group (Figure 3B,E,H). Glycosyltransferases with risk characteristics were significantly different between the high-risk and low-risk groups. GnT-IVb (N-Acetylglucosaminyltransferase-IVb, encoded by *MGAT4B*) was expressed highly in the low-risk group, while CHPF2 (Chondroitin sulfate glucuronyltransferase, encoded by *CHPF2*), EXTL2 (Exostosin-like 2, encoded by EXTL2), and POMT2 (O-mannosyltransferase 2, encoded by *POMT2*) were highly expressed in the high-risk group (Figure 3C,F,I). The progression-free survival analysis of the high- and low-risk groups found that the survival rate of the high-risk group was lower than that of the low-risk group (Figure 3J). In addition, the area under the ROC curve further confirmed the predictive power of the prognostic model, with AUC values of 0.609, 0.616, and 0.548 for 1, 3, and 5 years, respectively.

### 3.3. Verification of the Prognostic Ability of Four Cuproptosis-Related Glycosyltransferases in COAD Patients

**Figure 4:**
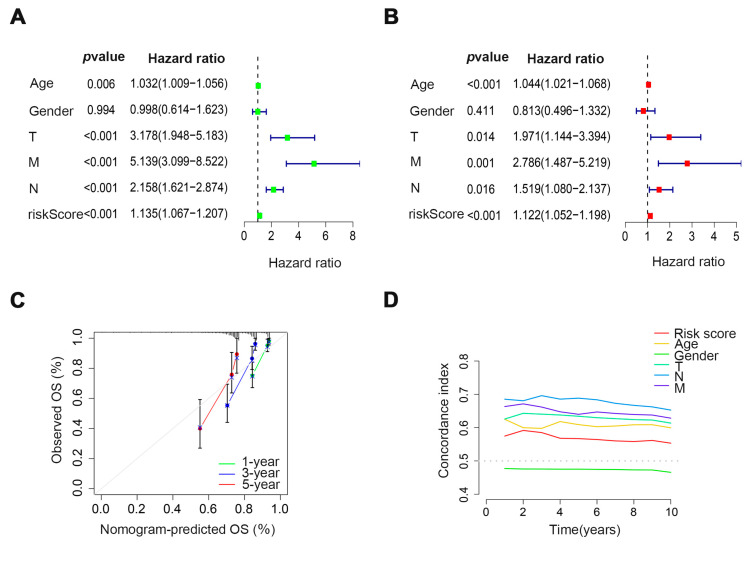

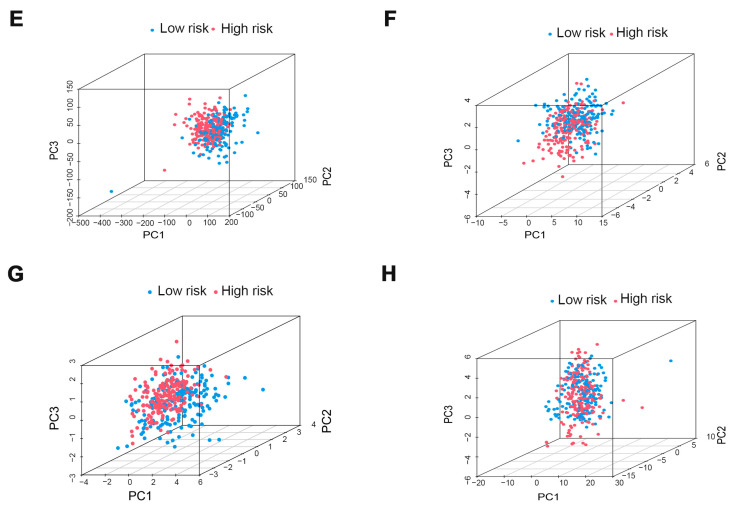
The prognostic ability of four cuproptosisrelated glycosyltransferases in COAD patients was verified. Univariate (**A**) and multivariate (**B**) COX regression analysis showed that risk score could be utilized as a prognostic factor. (**C**) The calibration curve predicted the patient’s OS at 1, 3, and 5 years in all samples. (**D**) C-index curves were used to assess the discrimination between the risk scores and other clinical features at different time points. PCA shows the sample distributions of all genes (**E**), cuproptosis-related genes (**F**), glycosyltransferases (**G**), and four cuproptosis-related glycosyltransferases (**H**).

In order to investigate the predictive value of cuproptosis-related glycosyltransferases in COAD patients, univariate and multivariate COX regression analyses were utilized. The results revealed a substantial correlation between the risk score and survival rates of COAD patients (Figure 4A,B), suggesting that the risk score can be utilized as an independent prognostic trait to forecast the prognosis traits of COAD patients. The 1, 3, and 5-year survival calibration curve for COAD patients also further demonstrated the accuracy of prognostic predictive models (Figure 4C). The C-index curve also showed that the risk score could be used as an indicator of patient prognosis compared to other risk factors (Figure 4D). Next, we examined the differences between the high-risk group and the low-risk group using PCA. The findings revealed that there was no difference in all genes, cuproptosis-related genes, and glycosyltransferases (Figure 4E–G). However, the COAD patients were split into two distinct groups according to the expression of the cuproptosis-related glycosyltransferases (Figure 4H). Therefore, the prognostic model can help predict the prognosis of COAD patients and classify patients with different clinical features into high-risk and low-risk groups.

### 3.4. Potential Function of Cuproptosis-Related Glycosyltransferases

**Figure 5:**
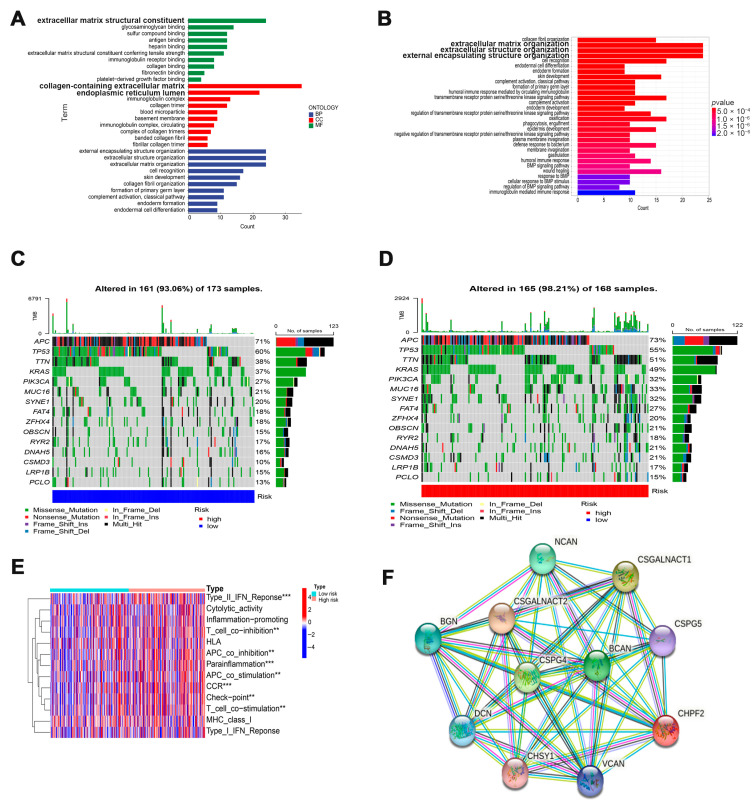

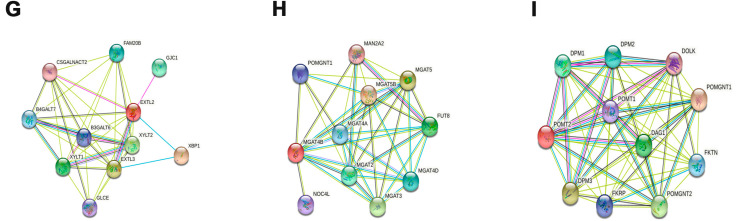
The functional analysis of four cuproptosis-related glycosyltransferases is shown. GO (**A**) and KEGG (**B**) functional enrichment analyses were performed for differentially expressed genes in the high and low-risk groups. The waterfall plot shows the frequency of mutations in the low-risk group (**C**) and the high-risk group (**D**). Difference analysis of the immune function between the high and low-risk groups (**E**). Prediction of the interacting proteins for cuproptosis-related glycosyltransferases CHPF2, EXTL2, GnT-IVb, and POMT2 by the STRING website (**F**–**I**).

We compared the gene expression in the high-risk and low-risk group COAD patients and obtained 299 differentially expressed genes. Then, we analyzed the differential genes using GO and KEGG functional enrichment assays. The GO analysis results showed that these differential genes were mainly enriched in collagen-containing extracellular matrix, extracellular matrix structural constituents, and endoplasmic reticulum lumen (Figure 5A). The KEGG results showed that the different genes were highly enriched in extracellular matrix organizations, extracellular structure organizations, and external encapsulating structure organizations (Figure 5B). The results showed that among the 15 genes with the highest mutation frequency, the gene mutation rate of the high-risk group, except APC, was higher than that of the low-risk group (Figure 5C,D). To find differences in the immune function between the high and low-risk groups, we further analyzed the immune pathways, and the results showed that the highly enriched pathways mainly included the Type II IFN response, parainflammation, and CCR (Chemokine Receptor) (Figure 5E). These results showed that cuproptosis may be associated with tumor immunity. Four cuproptosis-related glycosyltransferase interaction proteins, including CHPF2, EXTL2, GnT-IVb, and POMT2 were predicted using the STRING website, and the results revealed that most of these glycosyltransferase-interacting proteins were proteoglycans or other glycosyltransferases (Figure 5F–I). However, the results showed an interaction between EXTL2 and XBP1 (X-Box Binding Protein 1), a key protein in the endoplasmic reticulum stress pathway, suggesting that EXTL2 may play an important role in endoplasmic reticulum stress. *XBP1* is located at 22q12.1 on chromosome 12 and consists of six exons. It encodes a 376-amino acid protein, XBP1s, and a 261-amino acid protein, XBP1u [21]. It has been shown that XBP1 binds to cAMP response element (CRE) sites or CRE-like elements in the promoters of target genes [22], which are involved in metabolism, cell proliferation [23,24], and ER stress [25]. XBP1 expression is associated with the prognosis of several cancers, including breast cancer [26] and lung adenocarcinoma [27]. The above results suggest that cuproptosis-related glycosyltransferases not only play a role in tumor immunity but may also be involved in endoplasmic reticulum stress, which is necessary for cell survival.

### 3.5. The Role of Risk Scores in Drug Therapy

**Figure 6:**
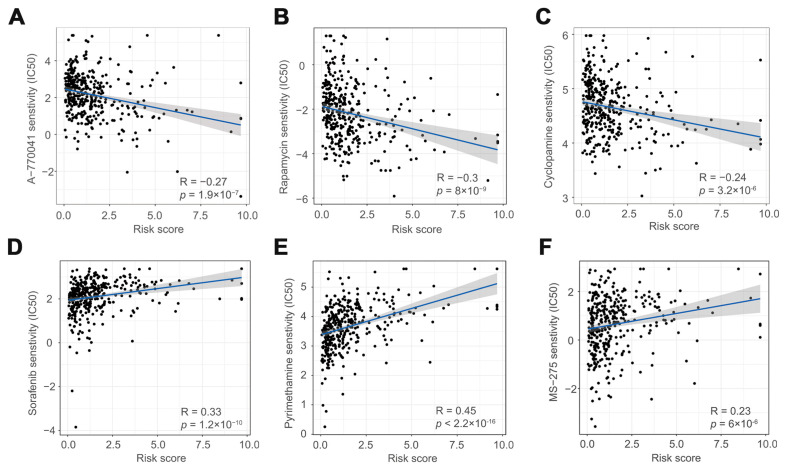

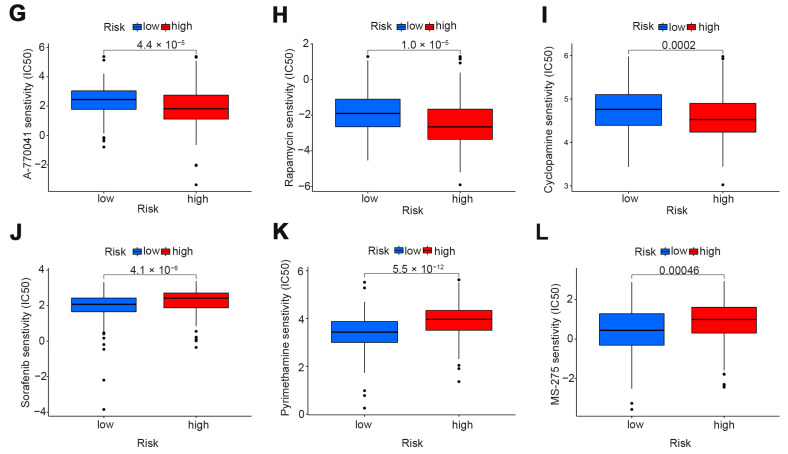
A-770041 (**A**,**G**), rapamycin (**B**,**H**), cyclopamine (**C**,**I**), sorafenib (**D**,**J**), pyrimethamine (**E**,**K**), and MS-275 (**F**,**L**) drug correlation and sensitivity analysis.

To explore the significance of the prognostic models in drug therapy, we assessed patients with COAD with different risk scores and sensitivity to various anticancer drugs. The statistical results showed that there were significant differences in drug sensitivity between the high-risk and low-risk groups. Patients in the low-risk group were more sensitive to A-770041, rapamycin, and cyclopamine than those in the high-risk group (Figure 6A–C,G–I). However, patients in the low-risk group were less sensitive to sorafenib, pyrimethamine, and MS-275 than those in the high-risk group (Figure 6D–F,J–L). These results imply that risk scores in prognostic models can assist in the treatment and drug selection of patients with COAD.

## 4. Discussion

In this study, we explored the correlation between cuproptosis and glycosyltransferases and constructed a prognostic model using cuproptosis-related glycosyltransferases, which had not been explored before. In addition, we further analyzed the functions of the four glycosyltransferases involved in the model construction and found that they may be involved in a variety of biological functions, including tumor immunity, endoplasmic reticulum stress, and so on.

Glycosylation of proteins alters their biophysical properties, function, distribution, and retention at the plasma membrane and modulates cell behavior, cell interactions, specific ligand–receptor interactions, and immune recognition [28,29,30,31]. Moreover, studies have shown that glycosyltransferase and glycosylation levels are altered during inflammatory conditions, tumorigenesis, and metastasis [32]. EXTL2, one of three Ext-like genes homologous to EXT1 and EXT2 in the human genome, encodes an N-acetylhexosaminyltransferase. It also plays an important role in the biosynthesis of glycosaminoglycans [33]. Exostosin-like 2 (EXTL2) has dual catalytic activity in vitro. Enzymatic analysis showed that the enzyme could be used as both -GlcNAc and -GalNAc glycosyltransferase synthesis linker. GnT-IVb (encoded by *MGAT4B*) is an enzyme that catalyzes the formation of the β1,4-GlcNAc branch in N-glycans. The isoenzymes GnT-IVa (N-Acetylglucosaminyltransferase-IVa) and GnT-IVb (*MGAT4B*) catalyze the synthesis of the β1,4-GlcNAc branch in N-glycans both in vitro and in cells. GnT-IVa and GnT-IVb prefer different glycoproteins. Notably, GnT-IVb acted more efficiently on glycoproteins bearing an N-glycan premodified by GnT-IV [34]. Because the expression levels of each N-glycan branch on specific glycoproteins are highly correlated with various diseases, such as cancer, diabetes, and Alzheimer’s disease [35], GnT-IVb may be a potential drug target to combat these diseases. They may act by participating in matrix interactions, glycosphingolipid metabolic pathways, and immune responses.

Furthermore, *POMT2*, encoding O-mannoyltransferase 2 (POMT2), is one of the pathogenic genes of α-DGP (Alpha-dystroglycanopathy). POMT2 catalyzes the first step in the biosynthesis of α-DG O-mannosylated glycans by transferring mannose to serine or threonine residues [36]. Mutations in *POMT2* cause Walker–Warburg syndrome [37]. In recent decades, the activation of programmed cell death has become an intense research focus in cancer research. [38]. Cuproptosis is a new type of cell death, different from any previously known cell death type, which also suggests that cuproptosis may lead to new solutions for cancer treatment. According to a previous study, glycosylation of ATP7A, one of the key enzymes of cuproptosis, promotes the plasma membrane localization and copper sensitive trafficking pattern [39], which implied the possibility that glycosyltransferases regulate cell death by modifying cuproptosis-related genes.

Our study also had some limitations. First of all, our model was created using the TCGA database without clinical sample verification. In addition, although four glycosyltransferase genes (*CHPF2*, *EXTL2*, *MGAT4B*, and *POMT2*) showed strong prognostic ability in this study, they were limited to a single database. A large number of different data points are lacking for further verification. In conclusion, we screened four cuproptosis-related glycosyltransferases associated with the prognosis of COAD patients and further evaluated their ability to predict survival and prognosis. This study establishes the correlation between cuproptosis and glycosyltransferase in COAD, which may provide a new perspective for the study of cuproptosis and a new strategy for the treatment of COAD patients.

## Data Availability

The data are contained within the article.
